# Preparation of MoO_3_/MoS_2-E_ composite for enhanced photoelectrocatalytic removal of antimony from petrochemical wastewaters

**DOI:** 10.55730/1300-0527.3450

**Published:** 2022-07-21

**Authors:** Sevil AKÇAĞLAR

**Affiliations:** Department of Mechanical Engineering, Faculty of Engineering, Dokuz Eylül University, İzmir, Turkey

**Keywords:** Photoelectrocatalytic, MoO_3_, MoS_2-E_, antimony, XRD, wastewater

## Abstract

By doping of MoO_3_ to MoS_2-E_, MoO_3_/MoS_2-E_ composite was produced to treat the antimony (Sb^+5^) from raw petrochemical industry wastewater. The effects of increasing MoO_3_/MoS_2-E_ composite concentrations (0.01, 0.06, 0.50, 1.20, and 6 mg/L), times (5 min, 10 min, 20 min, 60 min, 80 min), and simulated sun light powers (2, 15, and 26 mW/m^2^) on the removal of Sb^+5^ was researched. According to X-ray diffraction (XRD), MoS_2-E_ exhibited a pure hexagonal structure with peaks at 2Ɵ data of 15.56, 33.78, 40.59, and 61.43 cm^−1^ while MoO_3_ peaks showed similar configuration with the orthorhombic stage. X-ray photoelectron spectroscopy (XPS) was used to analyze the chemical composition. After Sb^+5^ removal, the additional MoO_3_ peaks were determined at 680, 967, and 997 cm^−1^. XPS spectra showed that after an oxidation period, “MoS_2-E_” was generated. Binding energy analysis showed that Mo^5+^ ions were produced from the partial transformation of MoO_3._ The MoO_3_ exhibited a vertical stacking on the MoS_2-E_. The filtered MoS_2-E_ graph and relevant fast Fourier transfer pictures showed octahedral phase containing a proton. Field emission scanning electron microscopy analysis results showed that nano MoO_3_ exhibited a nanobelt structure. The maximum 10 mg/L Sb^+5^ removal was 93% at 1.20 mg/L MoO_3_/MoS_2-E_ composite concentration at pH = 9 after 20 min at 15 mW/m^2^ simulated sunlight power via photoelectrocatalysis while the maximum Sb^+5^ removal via adsorption was detected as 80% for the same operational conditions in unilluminated conditions.

## 1. Introduction

Antimony (Sb^+5^) is generally present in wastewaters and in groundwater at high concentrations [1, 2]. Sb^+5^ is known to produce carcinogenic and toxic effects in the ecosystem [1, 2]. The content of petroleum pollutants is one of the parameters evaluating the water quality and its impact is increasingly important [1]. At present, the research on petroleum pollutant studies have been reported that water pollution was relatively serious in the river entrance into the lake, and the content of petroleum pollutants exceeded the limit values. Major sources of Sb^+5^ pollution are mining and processing activities coming from the mining waste rock, smelting waste, tailings dam, and underground tunnel wastewater [2]. Therefore, antimony has been listed as a priority pollutant by the US Environmental Protection Agency and the European Union [1, 2]. So far, the researchers are mainly focused on the leaching of some resources, and there are few studies on the characteristics of metal pollution in different functional mining zones. Previous studies have shown that the exploitation and smelting of antimony result in very serious Sb pollution in the soils of the surrounding mining areas, and antimony smelting slag is an important source of Sb pollution in nearby farmland soils [1, 2]. Some advanced removal technologies such as photocatalytic oxidation and photoelectrochemical removal processes are effective to remediate the Sb^+5^ from contaminated water, wastewaters, and ecosystems [1, 2]. Sb^+5^ is used in the production of flame retardants, polypropylene, and polyamide productions in petrochemical industry together chromium and lead.

Molybdenum drioxide (MoO_3)_ / molybdenum disulfide (MoS_2_) composite is a core-shell nanorod with high surface properties [3–5]. Molybdenum disulfide (MoS_2_) contained some active edge regions and exhibits crystallite properties [6]. High photooxidation rates can be determined by releasing of active sites conducting the two electron phases to an electron phase of MoS_2_ (MoS_2-E_) [6]. With its active regions and electronic conductivity, MoS_2-E_ particle improves the yield of hydrogen generation rates. Its weak light absorption increases the photooxidation rates [7]. MoO_3_ has low conduction property and is an open-structure stable nanomatter. Therefore, MoO_3_ not only exhibits photophysical and photochemical properties, but advise well the diffusion of ions [8]”ISSN”:”15287505”,”abstract”:”The charge transfer characteristics of metastable-phase hexagonal molybdenum oxide (h-MoO3. By doping of MoS_2-E_ with MoO_3_ a heterostructure nanocomposite was produced. This nanocomposite minimizes the regeneration of electrons during the mobilization of carriers. As a result, high photoelectrochemical yields was obtained [9]. Hwang et al. investigated the rhodamine blue (RhB) dye removal at increasing MoO_3/_MoS_2_ composite concentrations [4]. With an MoO_3_/MoS_2_ nanorod concentration of 45 mg/L, an RhB adsorption yield of Q_max_ = 326.8 mg/g was detected. Furthermore, Hwang et al. found that MoO_3_/MoS_2_ composite exhibits high-performance photocatalytic degradation ability for RhB dye [4]. Zhou et al. found 86% Pb^+2^, 87% Au^+3^, and 85% methylene blue removal with 46 mg/L MoO_3_/MoS_2_ composite [5]. Hwang et al. found 98% photocatalytic yield for methylene blue dye by incorporation of TiO_2_ to (MoO_3_)/MoS_2_ composite [4]. Saadati et al. found a photoluminescence and cophotocatalytic oxidation feature by using a heterojunction (MoO_3_)/MoS_2_ nanocomposite [10]. Zhao et al. found high photocatalytic activity by doping of 1T/2H-MoS_2_ **to** MoO_3_ **nanowires** [11]. Chui and Sun produced tremella-like molybdenum disulfide (MoS_2_), Mo trioxide (MoO_3_)/MoS_2_ and MoO_3_ nanoplates by using the pristine MoS_2_ nanosheets as the precursor [12]. With this nanocomposite 96, 90% methylene blue (MB) adsorption was detected at MB and nanocomposite concentrations of 10 mg/L and 20 mg/L, respectively. Gusain investigated the adsorption capability of molybdenum sulfide (MoS_2_)/thiol-functionalized multiwalled nanocomposite in the mining wastes [13]. Eighty-nine percent Pb^+2^ and 79% Cd^+2^ yields were detected using 9 g/L MoS_2_/thiol nanocomposite from the industrial mining wastes. Sheng et al. found >95% 2,4,6-trichlorophenol (TCP) and 94% photodegradation efficiencies for bio refractory halogenated organic compounds by Fe^+2^ doped MoS_2_ photocatalyst [14]. Chandrabose et al. found 89% total removal for dyed pollutants by integrated an adsorption-photocatalysis technique using 2-D MoS_2_/TiO_2_ nanocomposite [15]. Li et al. ( 2020) found a fast degradation for 10 mg/L RhB solution with a yield of 92% and a degradation rate of ~0.138 min^−1^ within 20 min under visible light (>420 nm) irradiation using a novel ternary MoS_2_/MoO_3_/TiO_2_ **composite** by photocatalysis [16]. Chen et al. found a high adsorption ability for porous MoO_3_/MoS_2_ in the removal of RhB dye via simultaneous adsorption and photodegradation [17].

As aforementioned and summarized in a recent literature survey, it was observed that MoO_3_/MoS_2_ nanocomposite was used extensively in the removal of some dyes and heavy metals in recent years. Although multiple data were obtained for MoO_3_/MoS_2_ composite throughout removal of dyes and some pollutant removal in recent literature, no study undertaking the removal of Sb^+5^ using the MoO_3_/MoS_2_ composite was found. Furthermore, no data was found about the adsorption and photocatalytic properties of the Sb^+5^ removal from a petrochemical industry wastewater with MoO_3_/MoS_2_ nanocomposite. The effects of the sun light powers, pH, and ionic strengths on the removal of Sb^+5^ were not studied before using MoO_3_/MoS_2_ composite.

The aim of the study was to treat the Sb^+5^ from a raw petrochemical industry wastewater via photoelectrochemical process. The effects of increasing MoO_3_/MoS_2-E_ composite concentrations (0.01, 0.06, 0.5, 1.2, and 6 mg/L), times (5 min, 10 min, 20 min 60 min 80 min), simulated sun light powers (2 mW/m^2^ in winter at 12:00, 15 mW/m^2^ in spring at 12:00, and 26 mW/m^2^ in summer at 12:00), increasing pH (4–9) and ionic strengths (HCO_3_^−^ for 0 and 9.0 mg/L at pHs 6 and 9; NO_3_^−^, Cl^−^, CO_3_^2−^, SO_4_^2−^, PO_4_^3−^, and SiO_3_^2−^ for 0, 0.5, and 0.7 mg/L at pHs 6 and 9) on the removal of Sb^+5^ were investigated. Meanwhile, the effects of increasing MoO_3_ composite concentrations (0.05, 0.08, 0.10, 0.15, 0.20, and 0.30 mg/L) in the MoO_3_/MoS_2_-_E_ on the Sb^+5^ yields were researched. Furthermore, the adsorption of Sb+5 to MoO_3_/MoS_2_ was also studied by using similar operational conditions in unilluminated conditions.

## 2. Materials and methods

### 2.1. Preparation of MoS_2-E_

MoS_2-E_ (Merck, Darmstadt-Germany) powder with particle sizes between 3 and 9 μm was used and was mixed with ethanol.

### 2.2. Preparation of MoO_3_/MoS_2-E_ composite

Firstly, 650 mg MoS_2-E_ (Merck, Darmstadt-Germany) and 38 mg MoO_3_ (Merck, Darmstadt-Germany) were mixed into a 250 mL glass flask. It was located in a sonicator for 7 h. It was then centrifuged at 8000 rpm for 60 min in a Hatch Lange centrifuge (Dusseldorf-Germany, 2018). The supernatant water was collected and it was transferred into a supercritical Merck CO_2_ incubator (Nüve, İstanbul-Turkey) with a heating jacket in a Gallemcamp autoclave (Roma-Italy). The autoclave was heated to 50 °C, and then CO_2_ was charged into the desired pressure (8 MPa). Then, the dispersed volume was collected [18]. In order to minimize the cost of the MoO_3_/MoS_2-E_ composite prepared under laboratory conditions, the most appropriate cheap and native chemicals were used.

### 2.3. Reactor configuration for photoelectrocatalytic removal of Sb^+5^

A 5-L photoelectrocatalytical quartz glass rector (Merck, Darmstadt-Germany) was used in this study. The efficiency of the electrodes during simulated sunlight irradiation was evaluated in a device containing three electrodes under simulated sunlight powers varying between 2 Mw/m^2^ and 26 mW/m^2^. Seven milligrams per liter sample was dispersed in methanol (Merck, Darmstadt-Germany) containing fluorine (Merck, Darmstadt-Germany) and suitable MoO_3_/MoS_2-E_ composite concentrations. The electrolyte was 0.3 M Na_2_SO_4_ (Merck, Darmstadt-Germany). The known-control electrode was Ag/AgCl (Gallemcamp, Roma-Italy) in saturated KCl (Merck, Darmstadt-Germany), and a Pt wire (Nüve, İstanbul-Turkey) was utilized for counter the electrode. The response was checked with simulated sunlight irradiation powers varying between 2 mW/m^2^ and 26 mW/m^2^ containing a KGS UV filter with a thickness of 2.5 mm (Sigma, St. Loius, Mo-ABD).

The Sb^+5^ photooxidation analysis was done in a 200 mL quartz glass reactor (Nüve, İstanbul-Turkey) using three electrodes with simulated sunlight containing a Horasan UV filter (Nüve, İstanbul-Turkey) at 460 nm at an OD of 0.89. The light power was adjusted to 2, 15, and 26 mW/m^2^ to simulate four seasonal conditions. Thirty-five milligrams per liter sample was dispersed in 6 mL 95% ethanol (Merck, Darmstadt-Germany). This process was maintained in dark during 80 min to reach steady-state conditions during the adsorption of the electrode [18]. The samples containing Sb^+5^ were taken from a raw petrochemical industry wastewater. The nanocomposite diameters were measured under an advanced three ocular light microscope (Bushman-Biotar, Poznan-Poland)

### 2.4. Adsorption study

For Sb^+5^ adsorption analysis, quartz glass reactors with volumes of 400 mL were used. They were stirred continuously with a magnetic stirrer at a velocity of 1600 min^−1^ under unilluminated conditions at room temperature. The same operational conditions utilized in the photocatalytic reactor were applied to the adsorption reactor.

### 2.5. Measurement of Sb^+5^

The concentration of Sb^+5^ was determined using an Optima 7300 DV inductively coupled plasma-optical emission spectrometer ( ICP-OES) to detect the correct concentrations of Sb ^+5^ [19]. The plasma and the auxiliary gas flows were 15 L/min and 0.2 L/min, respectively. The instrument contained a Meinhard concentric pneumatic nebulizer and a cyclonic nebulizer chamber attached to a peristaltic pump. Both are used to introduce the samples into the plasma. An axial view and a spectral line of 220.353 nm were used for Sb^+5^. For the preparation step, an ultrasonic bath was used. A 2 mL volume of a 10% HNO_3_ solution (v/v) was added to the petrochemical industry wastewaters containing Sb^+5^, and the samples were diluted to a final volume of 10 mL. The samples were then subjected to an ultrasonic bath for 20 min at 25 kHz and heated in a water bath for 1 h at 100 °C. Finally, the swabs were removed from the tubes, and the resulting solutions were analyzed by ICP OES. Data from the time-resolved ICP emission were accumulated during the construction phase of each chromatographic analysis. The values used for the eluent were 40 mM ethylendiaminetetraacetic acid (EDTA) at a flow rate of 1.0 mL/min. A 300 μL loop was used to manually inject the samples. The nebulizer flow was set to 0.7 L/min, reading time 1 s, RF power 1.2 kW. The accumulation of ICP-OES measurements was done in axial mode only to ensure the highest accuracy. The collected time-resolved emissions data was exported as text (CSV file), which was then integrated using the spreadsheet software Excel. Measurements of 1, 25, 50, 125, 250, 500, 1000, 2500, and 5000 mg/L were used to obtain the calibration curves. A linear model was chosen to fit the dependence of the total analyte count concentration. The limit of detection was stated as the concentration of analyte giving signals equivalent to three times the standard deviation of the blank plus the net blank intensity, for six independent replicates. Sb^+5^ recoveries for all analyses ranged from 98% to 99%.

Method detection limits (MDLs) were based on seven replicate measurements of a set of spiked calibration blanks. Analyte was added to each blank solution at concentrations estimated between 2 and 5 times the IDL. The MDL was calculated by multiplying the standard deviation of the seven replicate measurements by the appropriate Student’s t-test value according to: MDL = (S) × (t) where s is the standard deviation and t is the Student’s t-value, based on a 99% confidence level.

Both the Student’s t-value and the standard deviation are based on n-1 degrees of freedom (t = 3.14 for six degrees of freedom). In order to establish the system performance, wastewater samples were measured along with appropriate standard certified reference materials (CRM).

Accuracy was calculated as the difference between the measured and certified concentrations for the CRM. The results are presented in the tables below. The accuracy and precision showed that the developed method performs well. The method detection limits calculated were generally in the low μg/L (ppb) range for a majority of elements. The reproducibility of the measurement was generally better than 2%. The analysis of spectral interference check solutions did not show any interference with any of the analytical lines selected.

### 2.6. Calculation of q_e_ via adsorption and photocatalytic yields of Sb^+5^

Langmuir isotherm was used to calculate the Sb^+5^ amount attached on the surface of MoO_3_/MoS_2-E_ composite at equilibrium conditions, the concentration of adsorbed Sb^+5^ can also be described as follows [20]:


(1) $${\text{q}}_{\text{e}}=\text{K }{\text{q}}_{\text{max}}\;{\text{C}}_{\text{e}}/1+\text{K }{\text{C}}_{\text{e}}$$

where *q_e_* and K are the concentration of adsorbed Sb^+5^ and the adsorption equilibrium constant, respectively. Q_ax_ and C_e_ are the maximum adsorption capacity and the concentration of the Sb^+5^ compound in the samples, respectively [20].

Eq. (1) can be rearranged into a linear form as shown below (Eq. 2):


(2) $${\text{C}}_{\text{e}}/{\text{q}}_{\text{e}}=(1/\text{K }{\text{q}}_{\text{max}})+({\text{C}}_{\text{e}}/{\text{q}}_{\text{max}})$$

A graph of C_e_ / q_e_ against C_e_ would yield a straight line, which determines the adsorption capacity term, q_max_ (mg/g), from the slope (1/q_max_) as well as the adsorption equilibrium constant, K (L/mg), from the intercept (1/q_max_ K). The adsorption terms q_max_ and *K* relatively specify the tendency of the adsorbate to the surface of nanoadsorbent and explains the physical, chemical, and dynamic characteristic of a nanoadsorbent [20].

The adsorption and photocatalytic yields of Sb^+5^ were calculated as follows:

Adsorption yield = (Sb^+5^ concentration before adsorption – Sb^+5^ concentration after adsorption) / Sb^+5^ concentration before adsorption) × 100.

Photocatalytic yield = (Sb^+5^ concentration before photocatalysis – Sb^+5^ concentration after photocatalysis) / Sb^+5^ concentration before photocatalysis × 100.

### 2.7. Validation of the methods

The data collected from the adsorption and photocatalytic studies and Sb^+5^ analyses were performed in triplicate samples with standard uncertainties and standard deviations. The data given in all figures and tables were the mean values of these data. Two control reactor for photocatalytic and adsorption studies were operated without Sb^+5^. The MoO_3_/MoS_2_ composite were produced serially in the same time to prevent the possible differences between nanocomposites.

## 3. Results and discussion

### 3.1. X-ray diffraction (XRD) and Raman spectra of the MoS_2-E_, MoO_3_ and MoO_3_/MoS_2-E_ composite

**Figure 1:**
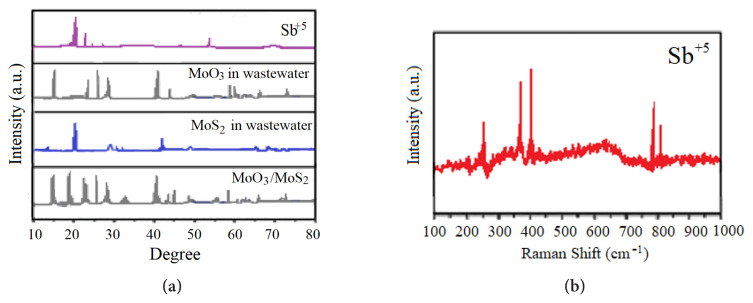
a) XRD distributions of Sb^+5^, MoO_3_, MoS_2-E_, and MoO_3_/MoS_2-E_ composite, b) Raman spectra of Sb^+5^.

The XRD patterns of the sample, MoS_2-E_, and MoO_3_ in wastewater are given in Figure 1a, while the Raman spectra of the sample are illustrated in Figure 1b. The peaks of the initial MoO_3_ in wastewater exhibited similar configuration with the orthorhombic stage of MoO_3_ (JCPSs no. 04-0509) [21]. During the treatment of Sb^+5^ in petrochemical wastewater, after MoS_2-E_ utilization, it was found that MoO_3_ showed a major crystal phase of hexagonal MoO_3_ in the MoS_2-E_ containing samples (JCPDs no. 22-0789). The differential peaks of this hexagonal MoO_3_ exhibited 2Ɵ values varying between 18.19° and 21.49° (Figure 1a) [22].

In Figure 1a, it is observed that MoS_2-E_ was generated and their patterns exhibited similarities with the XRD pattern. All different peaks in the XRD pathway can exactly show similarities with a pure hexagonal MoS_2-E_ stage (JCPDS NO. 38-1586). The major differential peaks at 2Ɵ data of 15.56, 33.78, 40.59, and 61.43 cm^−1^ can be attributed to reflections numbered (005), (109), (111), and (119), respectively. It is important to note that other special peaks were not detected. This showed that the produced composite exhibited high purity. Furthermore, differences in hills were detected since the crystal structure of the MoS_2-E_ changed [23, 24]. After Sb^+5^ removal, the additional MoO_3_ peaks determined at 680, 967, and 997 cm^−1^ as reported by Kumar et al. [25]i.e., MoO3-II, was a result of the topotactic phase transformation of hexagonal-MoO3 (h-MoO3. The presence of hills was found generally at 298, 696, 845, and 1002 cm^−1^ in this study. Furthermore, a novel Raman hill was detected at 232 cm^−1^ (Figure 1b). This can be explained by the presence of the photons produced by MoS_2-E_. As a result, two protons converted in a photon-containing step. Therefore, MoS_2-E_ transformed to pristine form (Figure 1b) [18]. This can be explained by the presence of the photons produced by MoO_3_/MoS_2-E_. As a result, two protons converted in a photon-containing step. Therefore, MoO_3_/MoS_2-E_ transformed to pristine form (Figure 1b).

### 3.2. X-ray photoelectron spectroscopy (XPS) analysis

**Figure 2:**
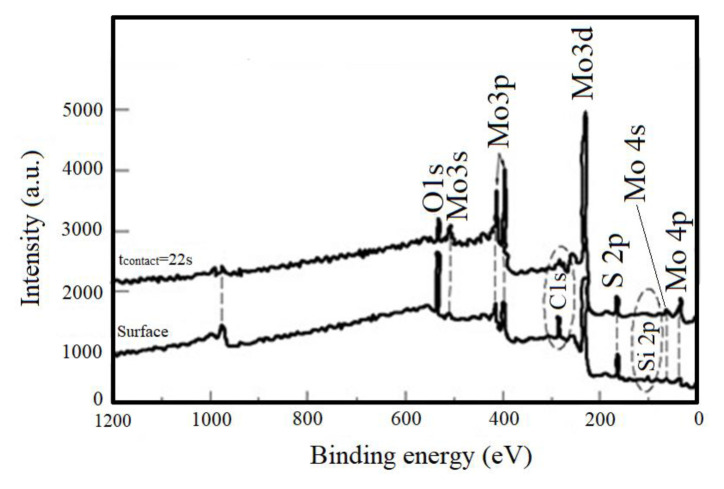
XPS survey spectra of MoS_2-E_.

C, Si, S, Mo, and O elements were found when XPS scanning of MoS_2-E_ was performed (Figure 2). The obtained Si 2p spectrum showed that binding energy was found at 103.3 eV for SiO_2_ [26]. The values found showed that the C, Si, and partial O elements were formed due to SiO_2_ and there were OH radicals absorbed on the MoO_3_/MoS_2-E_ surface.

Figure 3a shows the spectra of XPS Mo 3d, S 2p and O 1s given from MoS_2_ exposed to the photocatalytic mechanism. In the Mo 3d spectra, three peaks were detected as reported by Qi et al. and Yin et al. [27, 28]. These were Mo (IV) and Mo (VI) doublets and they corresponded to the Mo (VI) 3d_5/2_ at ~228.9 eV, Mo (V) 3d_3/2_ at ~236.8 eV, and a sum of Mo (VI) 3d_3/2_ and Mo (IV) 3d_5/2_ at ~231.9 eV were detected. Based on the position, area, and width of both Mo (VI) 3d_5/2_ and Mo (VI) 3d_3/2_ peaks, the Mo 3d spectrum was fitted to the two Mo (VI) and Mo (IV) peaks (Mo 3d_5/2_ and Mo3d_3/2_: 231.6, 236.5 eV; and 228.4, 231.6 eV).

The contribution of Mo 3d varied between 40% and 51% which is corresponding to MoO_3_ and MoS_x_O_y_, respectively [29, 30]. Two peaks were detected from the S 2p XPS spectra (Figure 3b). These separately corresponded exactly to MoS_x_O_y_ at 163.0 eV and to sulfuric acid at 169.1 eV as reported by Luther et al. and Manthiram and Alivisatos [31, 32].

The O 1s spectra shown a broad and asymmetric peak which should be a superposition of O 1s peaks with different chemical structures such as MoO_3_, MoS_x_O_y_, absorbed OH radicals and sulfonated contaminants ( Figure 3c) [33, 34].

**Figure 4:**
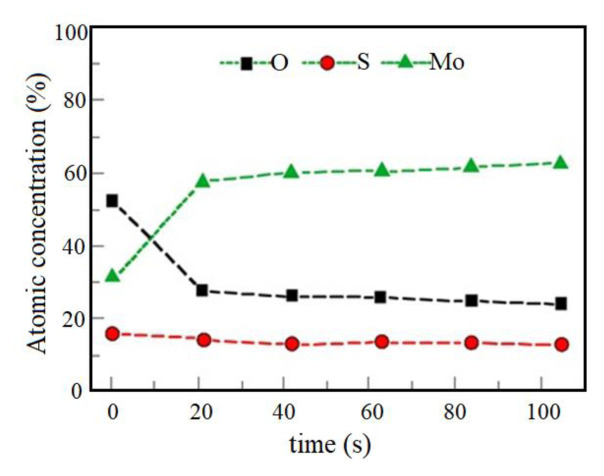
XPS profile of MoS_2-E_ before photocatalytic reaction.

Figure 4 shows the XPS profile of the MoS_2-E_ before being exposed to photoelectrocatalytic operation. The higher O and lower Mo concentrations were obtained at the surface, after which they were stable [35].

**Figure 5:**
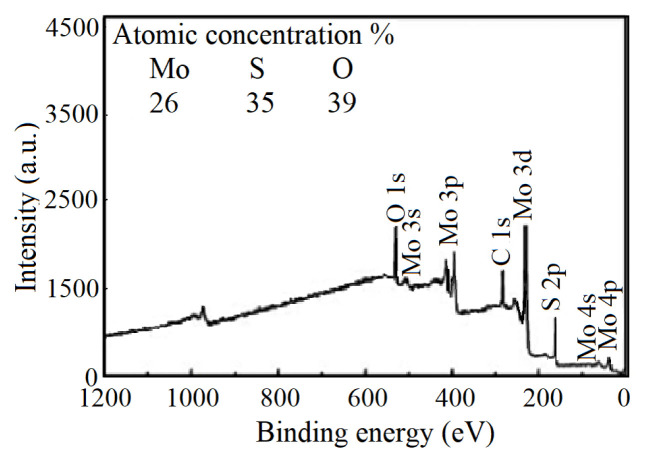
XPS profile of MoS_2-E_ after photocatalytic reaction.

Previous studies showed that [34, 36] SO and SO_2_ were already produced in the MoS_2_ as volatile products as a result of photocatalytic irradiation, and S losses was found [29, 36]. In this study, any significant variations not found in S concentration from the XPS survey of the MoS_2-E_ exposed to the photoelectrocatalytic operation. The element compositions obtained from the XPS survey spectrum showed that Mo, S, and O contents (with percentages of 26%, 35% and 39% respectively), normalized to 100% (Figure 5).

### 3.3. High-resolution transmission electron microscopy (HRTEM) analysis

**Figure 6:**
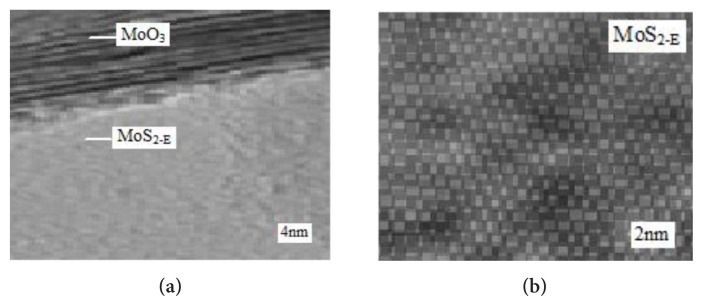
HRTEM images.

HRTEM was utilized to assess the morphology, the heterostructure and the lattice arrangement of the MoO_3_/MoS_2-E_ composite. A typical HRTEM image is shown in Figure 6a. The MoO_3_ exhibited nanostructure which has vertical stacking on the nano composite of MoS_2-E_. The filtered MoS_2-E_ graph and relevant fast Fourier transfer (FFT) picture is illustrated in Figure 6b. This was a trigonal (octahedral) phase of the MoS_2-E_ containing a proton in the comparison to the trigonal prismatic phase of the MoS_2-E_ containing two protons [27].

### 3.4. Energy-dispersive X-ray spectroscopy (EDS) analysis

**Figure 7:**
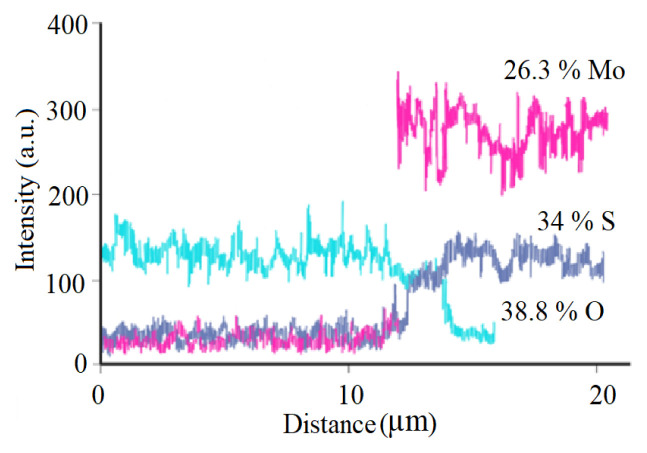
EDS line scan of MoO_3_/MoS_2-E_ composite.

**Figure 8:**
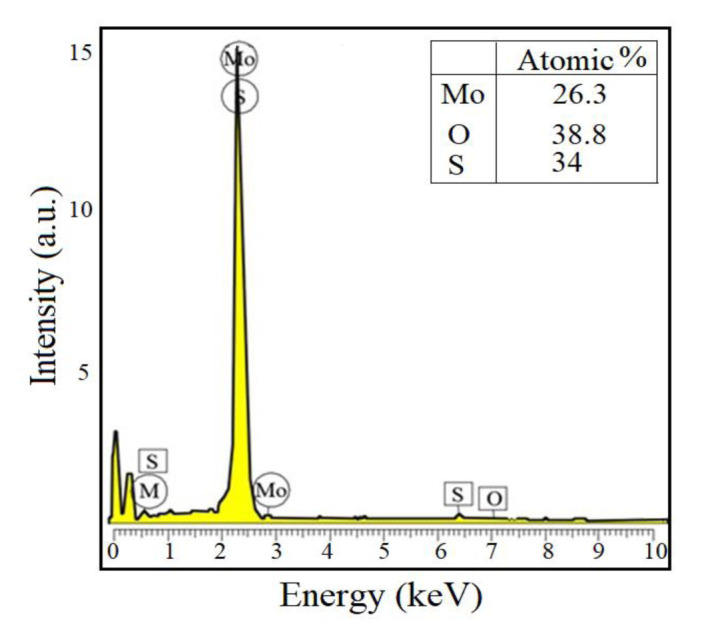
EDS spectrum of MoO_3_/MoS_2-E_ composite.

The EDS line scan of MoO_3_/MoS_2-E_ composite was given in Figure 7. As can be seen, the nanocomposite contains S, Mo, and O. The atomic percentages were clearly presented in Figure 8. It was shown that EDS analysis exhibited similarities with the data obtained from the XPS scan profile. The atomic percentages of Mo, S, and O obtained from Figure 8 were accounted as 26.3%, 34.9%, and 38.8% with a total score of 100%. These studies exhibit similarities with the study performed by Acerce et al. [37].

### 3.5. Field emission scanning electron microscopy (FESEM) analysis

**Figure 9:**
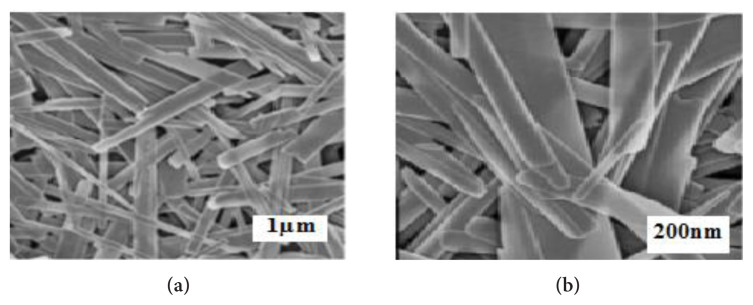
FESEM images.

The structure of the MoO_3_ and MoS_2-E_ nanoparticles produced under laboratory were investigated by the FESEM Analysis (Figures 9a and 9b). In general, the produced MoO_3_ nano material exhibited some nanobelt structure with a length of 3 nm (Figure 9a) [38].

**Figure 10:**
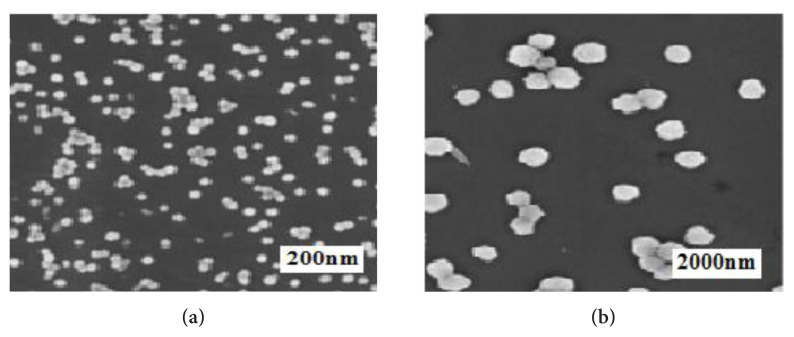
FESEM images.

Figure 10a shows the structure of MoO_3_ nanobelts. The results showed that the nanobelt generated contains a straight surrounding with mean widths varying between 88 and 439 nm. The MoS_2-E_ produced under laboratory condition is illustrated in Figure 10a. The nanoparticles produced have diameters between 42 and 89 nm.

Figure 10b exhibits an aggregate shape of the some MoS_2-E_ nanoparticles. These nanoparticles exhibited a rough surrounding. This originated from the stowing of the each one MoS_2-E_ layer. This result agrees with the elevated intensity of the (002) diffraction hill in XRD as reported by Sheng et al. and Tang et al. [38, 22].

### 3.6. Effect of MoO_3_/MoS_2-E_ concentrations on the removal of Sb^+5^ concentration via adsorption

The preliminary studies showed that the maximum adsorption yields of 10 mg/L Sb^+5^ was 73% with 0.80 mg/L MoO_3_/MoS_2-E_ composite after 28 min contacting time in the continuous studies (data not shown) in unilluminated conditions. As the MoO_3_/MoS_2-E_ composite concentration was increased from 0.01 mg/L up to 1.20 mg/L, the Sb^+5^ concentrations decreased to 2.0 mg/L with a Sb^+5^ yield of 80%. Table 1 showed the adsorption of Sb^+5^ and maximum adsorption capacity (q_e_). As the MoO_3_/MoS_2-E_ composite concentration was increased up to 3.0 mg/L; the Sb^+5^ removal remained stable in unilluminated conditions. The maximum Sb^+5^ yield was obtained at 1.20 mg/L MoO_3_/MoS_2-E_ composite concentration. However, at high MoO_3_/MoS_2-E_ composite concentrations, after an optimum Sb^+5^, the removal of Sb^+5^ decreased by the turbidity resulting in small amount of Sb^+5^ contacting with MoO_3_/MoS_2-E_ and low adsorption yields were detected. The mean diameter of MoO_3_/MoS_2-E_ composite generated under laboratory conditions was measured as 45 μm with an advanced light microscope. The batch studies performed with MoO_3_/MoS_2-E_ composites having low (30 μm) and high diameters (55 μm) did not exhibit significant differences in Sb^+5^ removal via adsorption process (82% and 83%) (data not shown).

**Figure 11:**
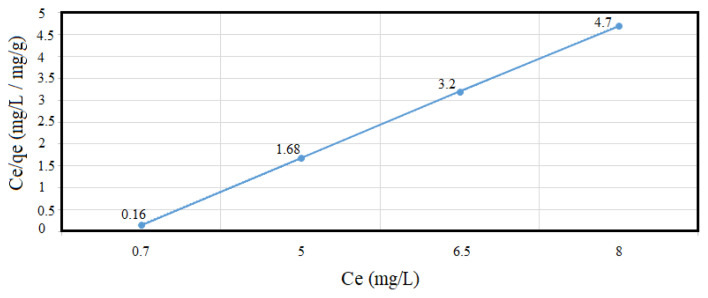
Langmuir isotherm by plotting C_e_ and C_e_/q_e_ values obtained from the adsorption study (q_max_ = 0.625 (mg/g) and K = 0.063 (L/mg)).

By plotting of C_e_ and C_e_ /q_e_ values obtained from the above table, it was found that the adsorption of Sb^+5^ on MoO_3_/MoS_2-E_ is suitable to Langmuir isotherm with q_max_ = 0.625 (mg/g) and K values of 0.063 (L/mg) exhibiting a linear plot with r = 0.99 and regression coefficient of R^2^ = 0.999 ( Figure 11).

### 3.7. Effect of increasing MoO_3_/MoS_2-E_ concentrations on photoelectrocatalytic removal at simulated sunlight powers

The preliminary studies showed that the maximum Sb^+5^ (10 mg/L Sb^+5^) photoelectrocatalytic yields (88%) was obtained with 1 mg/L MoO_3_/MoS_2-E_ composite at a sunlight power of 15 mW/m^2^ after 24 min irradiation time in the continuous studies (data not shown). As the MoO_3_/MoS_2-E_ composite concentration was increased from 0.01 mg/L up to 1.20 mg/L, the Sb^+5^ concentrations decreased to 0.70 mg/L with a Sb^+5^ yield of 93% (Table 2). As the MoO_3_/MoS_2-E_ composite concentration was increased up to 6.0 mg/L, the Sb^+5^ removal remained stable. The max Sb^+5^ yields were obtained at 1.20 mg/L MoO_3_/MoS_2-E_ composite concentration. The increase of Sb^+5^ yield versus increasing MoO_3_/MoS_2-E_ composite is the production of numerous active sites, and resulting in increasing of hydroxyl radical production during photooxidation. However, at high MoO_3_/MoS_2-E_ composite concentrations, after an optimum Sb^+5^ photo electrocatalytic level, the removal of Sb^+5^ decreased by the turbidity resulting in small amount of sunlight power contact and low photoelectrocatalytical efficiencies [18]. The mean diameter of MoO_3_/MoS_2-E_ composite generated under laboratory conditions was measured as 45 micrometer with an advanced light microscope. The batch studies performed with MoO_3_/MoS_2-E_ composites having low (30 micrometer) and high diameters (55 μm) did not exhibit significant differences in Sb^+5^ removal (92% and 91%) (data not shown).

The Sb^+5^ removal efficiency increased from 20% to 93% when the MoO_3_/MoS_2-E_ composite concentration was increased from 0.01 mg/L to 1.20 mg/L. A further increase in the MoO_3_/MoS_2-E_ composite (3 mg/L) resulted in a stable level of Sb^+5^ removal yields. At the beginning, the increase is due to the increased surface area and to the numerous suitable phtocatalytic activated points in the surface MoO_3_/MoS_2-E_ composite. At high MoO_3_/MoS_2-E_ composite concentrations such as 6 mg/L, the negative charge of the of Sb^+5^ was agglomerat ed. At high Sb^+5^ concentration like 1.20 mg/L, the activated surface regions of MoO_3_/MoS_2-E_ composite sites charged excessively and produced OH radicals were at high level. Under these conditions, the activated surface regions can regenerate electrons fully and they do not lose their activity [39]. Nineteen milligrams per liter Cu^+2^ and 21 mg/L As^+3^ present in petrochemical industry wastewater were removed via photocatalysis under similar operational conditions with photodegradation yields of 87% and 86.5%. This showed that MoO_3_/MoS_2_-_E_ composite can be used as an excellent photocatalyst to remove the heavy metals from the industrial wastewaters.

### 3.8. Photoelectrocatalytic effects of increasing MoO_3_ concentrations in MoO_3_/MoS_2-E_ on Sb^+5^ yields

As the MoO_3_ concentrations were increased from 0.05 mg/L up to 0.1 mg/L, the Sb^+5^ yields increased from 45% up to 92% under **s**unlight (Table 3). Further increase of MoO_3_ to 0.15 mg/L and to 0.30 mg/L did not improve the Sb^+5^ removal concentration. At these MoO_3_ levels, the photoelectrocatalytic removal of Sb^+5^ occurred via electron transferring of the electrodes under sunlight irradiation. The results showed that further increase of MoO_3_ concentration to 0.15 and to 0.30 mg/L affect negatively the Sb^+5^ yields. The reason for this is that the optimum amount of MoO_3_ increases the number of active sites on the photocatalyst surface, which, in turn, increase the number of hydroxyl radicals (OH^•^). However, excessive amounts of MoO_3_ can retard the photocatalysis process because of excess amount of dopants, resulting in same Sb^+5^ yields.

The chemicals used in the preparation of the MoO_3_/MoS_2-E_ was combined as low as to determine the optimum operational conditions to reach maximum removal both for adsorption and photooxidation processes under steady-state conditions. In these removal mechanisms, it is important to use the minimal concentrations and ratios of the reagents to develop the nanoadsorbent or nanocomposite. A cost analysis was performed to determine the MoO_3_/MoS_2-E_ spent. In order to remove 45 mg/L Sb^+5^ from 1 m^3^ petrochemical wastewater via photocatalysis under sun light, the cost was found as 0.6 Euro (data not shown).

### 3.9. The effect of photooxidation times on the photoelectrocatalytic removal of 10 mg/L Sb^+5^

The maximum Sb^+5^ yield was obtained as 93% after 20 min photoelectrocatalytic contacting time for the operational conditions given above (Table 4). The lowest Sb^+5^ photoelectrocatalytic yield (30%) was obtained at 5.0 min photooxidation time. An increment of time from 5.0 min up to 20 min increases the Sb^+5^ removal efficiency since the number of active places increases the removal yield of Sb^+5^.

### 3.10. Effect of simulated sunlight powers on the removal Sb^+5^ concentration at 1.20 mg/L MoO_3_/MoS_2-E_ composite

As shown in Table 5, an increase in sunlight power from 2 mW/m^2^ to15 mW/m^2^ and to 26 mW/m^2^, the removal efficiency of Sb^+5^ increased from 70% to 88% and to 98%. The electron production rate varied depending on increasing sunlight powers. At high solar power, higher electron conduction occurred [40]. More electron carrying Sb^+5^ can be photooxidated easily. The effect of sunlight power on the removal of the Pb^+5^ showed that the solar power was very effective in the treatment of Sb^+5^ (Table 5). The studies under UV exhibited superior performances (data not shown) since the electrons from UV exhibited higher entrance on the surface of the MoO_3_/MoS_2_ composite as reported by Prado et al. [41].

### 3.11. Effect of Sb^+5^ doses on the photocatalysis of increasing MoO_3_/MoS_2-E_ composite doses

**Figure 12:**
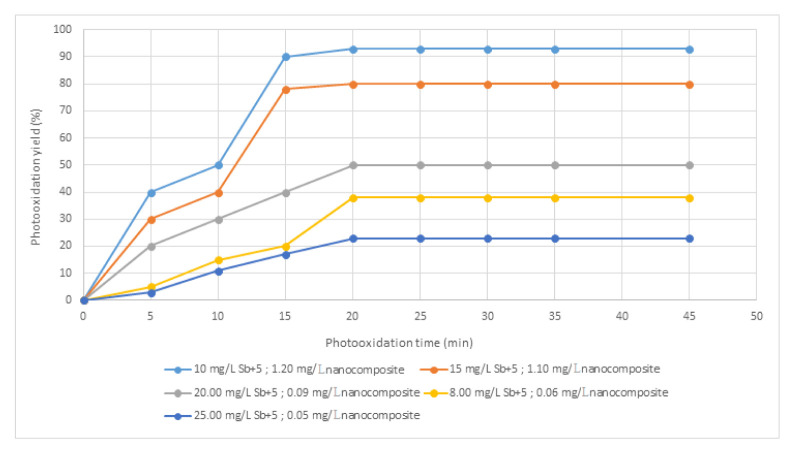
Effect of increasing Sb^+5^ and MoO_3_/MoS_2-E_ composite doses on the photocatalytic yield of Sb^+5^.

Figure 12 shows the photocatalysis results at increasing Sb^+5^ and nanocomposite doses. The maximum Sb^+5^ photooxidation yield (95%) was observed at 10 mg/L Sb^+5^ concentration where the MoO_3_/MoS_2-E_ concentration was optimum (1.20 mg/L) at all studies of this research. At very high Sb^+5^ concentrations (25 mg/L), the photooxidation rates decreased since the acidified groups OH• ions decreased during photooxidations as reported by Qi and Pichler (2016) [42]adsorption behavior and other chemical properties are similar to that of arsenic (As.

### 3.12. Effect of pH on the photooxidation Sb^+5^ at 1.20 mg/L MoO_3_/MoS_2-E_ concentration

In this step of the study, in order to detect the influence of pH on photooxidation yields of Sb^+5^, studies were performed at six pH values (4, 5, 6, 7, 8, and 9). As shown in Table 6, during the photooxidation of Sb^+5^, the yield elevated with rising of pH. The removal efficiency of Sb^+5^ was measured as 45% at pH = 4 after 20 min contacting time. As the pH was increased to 5, no significant increase in Sb^+5^ removal was detected. The Sb^+5^ photooxidation yields was detected as 47% at this pH after 20 min. Then the pH increased to 6 and 9, respectively. The Sb^+5^ photooxidation yields were measured as 72% and 74%, respectively, in the aforementioned pH levels. The maximum Sb^+5^ photooxidation yield was detected as 81% after 20 min at pH = 8. The photoremoval of Sb^+5^ increased immediately after 10 min and reached steady state after 20 min at pH = 9 with a maximum Sb^+5^ photodegradation yield of 97%. Under alkaline conditions, the Sb^+5^ photoremoval is better than a neutral and acidic condition as reported by Qi and Pichler [42]adsorption behavior and other chemical properties are similar to that of arsenic (As. With increasing pH, the Sb^+5^ mainly exists in negative form, and the electron intensity of Sb^+5^ increased. This is the recommendation for the formation of reactive oxidative species resulting in increased photodegradation. O_2_ and O_2_^−^ radical species were generated. The deprotonated Sb^+5^ absorbed photons and to be excited to a triplet [43]adsorption behavior and other chemical properties are similar to that of arsenic (As. Since point zero charge of the MoO_3_/MoS_2-E_ composite catalyst surface was measured as 8.9, the photodegradation rates decreased under acidic and neutral pH as performed by Li et al. [adsorption behavior and other chemical properties are similar to that of arsenic (As9].

Usually, the surface of MoO_3_/MoS_2-E_ is negatively charged and the primary mechanism in adsorption could be ion exchange which happens between Sb^+5^ ions and the cations on MoO_3_/MoS_2-E_ surface [44–47]. Due to the stronger electrostatic attraction, Sb^+5^ could first approach MoO_3_/MoS_2-E_ surface and replace H^+^, and then form a Pb-S complexation with one or two S atoms, depending on the abundance of Sb^+5^ ions [47]. The formation of Sb_2_S_5_ complexation is supported by the reaction generated in the surrounding of MoO_3_/MoS_2-E_ [48]. At the top layer of MoO_3_/MoS_2-E_, each S atom possesses a tetrahedral electron configuration because of sp3 hybridization. Three of the sp3 orbitals form MoO_3_/MoS_2-E_ –S bonds while the fourth is occupied by a pair of electrons [49–50]. In this theory, Sb^+5^ can accept the ion pair electrons on MoO_3_/MoS_2-E_ surface due to its empty 6p orbitals and form stable coordinate covalent bonds [51]. Similarly, both electrostatic attraction and Sb^+5^ – sulfur complexation contribute to the adsorption of Sb^+5^ ions on s MoO_3_/MoS_2-E_ surface.

### 3.13. The effect of HCO_3_^−^ on photoelectrocatalytical removal of Sb^+5^

**Figure 13:**
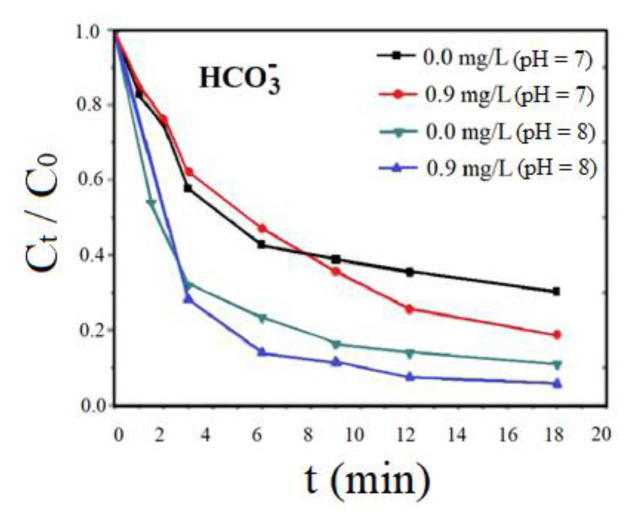
The influence of HCO_3_^−^ on the photo-oxidation of Sb^+5^ (C_0_ 10 mg/L, after 20 min, at sunlight power of 15 W/m^2^ at pH 7.0 and 8.0).

HCO_3_^−^ is a rich anion in water and does not absorb light in solar irradiation. However, it can react with strongly oxidizing radicals such as OH•, SO_4_^−2^ to produce some carbonate radicals (CO_3_^−2^). Also, adding HCO_3_^−^ sometimes elevates the pH of the medium. This decreases the photochemical activity of the some metals [26]whereas hydroxyl radicals (HO. The pK_a1_ and pK_a2_ of H_2_CO_3_ are 6.39 and 10.38, respectively [43]whereas hydroxyl radicals (HO. At neutral pH, HCO_3_^−^ ratio was measured as 78%, while 22% of the HCO_3_^−^ is measured as carbon dioxide dissolved in wastewater. Addition of bicarbonate (between 0.0 and 0.9 mg/L HCO_3_^−^) did significantly affect the removal yields of Sb^+5^ at investigated pHs (7.0 and 8.0) [52]and investigates its adsorptive behaviors toward Sb(III. The Sb^+5^ photoelectrocatalytical removal elevated after 20 min of irradiation time when HCO_3_^−^ doses were increased from 0.0 mg/L to 0.9 mg/L at pH 7.0 and 8.0 ( Figure 13). Meanwhile, it is important to note that Sb^+5^ removal increased significantly at pH 8.0 when the HCO_3_^−^ dose was increased from 0.0 mg/L to 0.9 mg/L. This change can be attributed to high bicarbonate concentrations releasing selective CO_3_^−2^ [53, 54].

### 3.14. Effect of NO_3_^−^, Cl^−^, CO_3_^2−^, SO_4_^2−^, PO_4_^3−^ and SiO_3_^2−^ ions on photoelectrocatalytical removal Sb^+5^

It is clear that the adsorption performance, toxicity, mobility, and even bioavailability of Sb^+5^ will be greatly affected by competing and coexisting anions such as PO_4_^3−^, O_3_^2−^, SiO_3_^2−^ NO_3_^−^, SO_4_^2−^, and Cl^−^. From the results of previous investigations, it was learned that the adsorption of Sb^+5^ on ferric adsorbents was not affected much by NO_3_^−^, or CO_3_^2−^, Cl^−^ [23]. It has been proven in the most performed experiments that Sb^+5^ adsorption is not affected in iron-based adsorbents by SO_4_^2−^ [55, 56]. However, Zhu, on the contrary, proved that the presence of SO_4_^2−^ fully supports Sb^+5^ adsorption on zerovalent iron [57]. Zhang et al. found that SO_4_^2−^ does not allow Sb^+5^ adsorption on zerovalence iron [58]. Besides, Hu et al. also discovered that Sb ^+5^ adsorption on Fe-Cu binary oxide is more affected by SO_4_^2−^ than Sb^+3^adsorption [59]. PO_4_^3−^ is known to have an inhibitory effect on the adsorption of Sb(VI) and Sb(III) on ferric adsorbents. No study was found investigating the effects of the aforementioned ions on Sb^+5^ photooxidation yields in the presence of MoO_3_/MoS_2-E_ composite. The effects increasing of some ion concentrations on the of 10 mg/L Sb^+5^ at 1.20 mg/L MoO_3_/MoS_2-E_ composite concentration at 0.10 mg/L MoO_3_ concentration after 20 min photooxidation of Sb^+5^ were tabulated in Table 7 for pH 6 and 9.

The results showed that the PO_4_^3−^, SiO_3_^2−^ ions increased the photocatalytic removal of Sb^+5^ at all concentrations for both pH values. Probably these ions did not compete with Sb^+5^ for adsorption sites on the surface of MoO_3_/MoS_2-E_ composite at the beginning of photocatalytic oxidation process and this phenomenon improve the scavenging effects of these anions by increasing the OH radical production as reported by Santiago et al. [60]. On the other hand, as reported by Jianhong et al., it is possible to distinguish between inner-sphere and outer-sphere anion surface complexes. The inner-sphere complex is stronger than the outer-sphere complex and was not dependent on the ionic strength in the presence of PO_4_^3−^, SiO_3_^2−^ ions [60]. When the PO_4_^3−^, SiO_3_^2−^ ion concentrations increased to 0.5 and 0.7 M at pH = 9.0, the Sb^+5^ might only form strong bond with MoO_3_/MoS_2-E_ and the adsorption of Sb^+^ on MoO_3_/MoS_2-E_ was increased by the changes in ionic strength as reported by Duo [61]. By contrast, the presence of NO_3_^−^, Cl^−^, CO_3_^2−^, and SO_4_^2−^ anions decrease the Sb^+5^ photooxidation yields due to nonscavenging effects of these anions by decreasing the OH radical production as reported by Santiago et al. [60]. The inhibition, promotion, competition, and synergistic interaction of coexisting/competing ions during Sb^+5^ photooxidation require further advanced research. These ions can compete for sorption of Sb^+5^ to outer MoO_3_/MoS_2-E_ surface at pH = 9.0 [61, 62]. Recent studies have shown that mineralization as well as disinfection of HCO^3−^, ClO^4−^, NO_3_^−^, SO_4_^2−^ and ions limits the activity of photocatalysts surface [63]. The effect of catalyst surface contamination with inorganic ions on photoactivity of TiO_2_ is explained using several mechanisms [64]. They include adsorption competition of photons at active sites and on the particle surface, direct interaction with the photocatalyst, scavenging of radicals and electron gaps, and the scanning effect of UV rays. Actually, these properties are displayed by SO_4_^2−^, NO_3_^−^, Cl, HCO^3−^, and PO_4_^3−^ ions. The mechanism of the photocatalysis inhibition by Cl^−^ and HCO_3_^−^ anions by scavenging of electron gaps and radicals are given by the reaction equations (3) and (4) [65].


(3) $${\text{Cl}}^{-}+\text{OH}•\to \text{Cl}•+\;{\text{OH}}^{-}$$

(4) $${\text{Cl}}^{-}+{\text{h}}^{+}\to \text{Cl}•$$

Furthermore, Cl^−^ ions cause inhibition of photocatalysis by TiO_2_, which can be explained by the preferential adsorption mechanism against the surface-bound OH^−^ ions. While reducing the amount of OH^−^ ions present on the TiO_2_ surface, substitution Cl^−^ ions cause increased recombination of electron-electron gap pairs [64–66]. The range of ionic Sb^+5^ species stabilizes as the pH changes, causing changes in adsorption behavior of Sb^+5^. The low adsorption of Sb^+5^ is likely due to competition for adsorption sites between hydroxyl ions, and for pH 4 to 6, Sb^+5^ is the [Sb (OH)^6−^] species [67]. For this reason, it is certain that the optimum pH value for adsorption at the initial stage of the photocatalytic mechanism is related to the zero charge point of MoO_3_/MoS_2-E_ in solution. The protonation reaction on the surface of the MoO_3_/MoS_2-E_ composite causes the surface to become positively charged, which ultimately increases the adsorption of Sb^+5^ ions due to the higher concentration of H^+^ ions in the reaction mixture, promoting the removal of Sb^+5^. On the contrary, if the pH is lower and higher than the zero charge pH, the MoO_3_/MoS_2-E_ composite surface and the adsorbate species will be negatively charged. Thus, the adsorption performance is affected through the process of electrostatic repulsion [68, 69]. In order to obtain good removal efficiency of Sb^+5^, the pH value should be kept below the pH_zpc_ of the iron adsorbents within the pH range of 8–9. For Sb^+3^, the neutral H_3_SbO_3_ species dominates over a wide pH range (2–10) [67]. Thus, a change in pH value has little influence on the removal efficiency of Sb^+3^ compared to Sb^+5^.

## 4. Discussion

The photocatalytic studies performed by some nanocatalysts showed that the removal of some heavy metals can be performed by a quick and short adsorption stage ending with photocatalytic reductions. In this study, both adsorption and photocatalytic removal of Sb^+ 5^ on MoO_3_/MoS_2-E_ were studied. Since the photocatalytic yield of Sb^+ 5^ is higher compared to adsorption capacity, detailed studies were performed with only photooxidation. Wang et al. found an adsorption of Ag^+^ on individual MoS_2_ surface and the other is the redox reaction that forms the segregated micrometer-sized metallic silver particles [70]. In this study, it was also found that some heavy metals like Ag^+1^, Au^3+^, and Hg^2+^ can be removed with a reduction–oxidation (redox) reaction between ionic metal species and MoS_2_ from water by two-dimensional MoS_2_ nanosheets suspended in aqueous solution [70]. Light also has been found to be an indispensable catalyst in the redox reaction of reduced metallic particles. Saadati et al. found that single-layer MoS_2_-MoO_3_ heterojunction nanosheets with lateral average dimensions of ~70 nm showed an excitation-dependent behavior along with visible-light-induced photocatalytic activity [10]. The existence of MoO_3_ domains with a broad light absorption due to the oxygen vacancies promoted the essential photocatalytic activity of single-layer MoS_2_ sheets by facilitating separation of photoexcited electron–hole pairs and preventing their fast recombination. Thus, eventually the photocatalytic activity was enhanced. The scavenging feature of oxidizing radical species given by the NO_3_^−^, SO_4_^2−^, ClO_4_^−^, and HCO_3_^−^ ions were also responsible for reducing the rate of degradation. NO_3_^−^ ion may block the active sites of the catalysts by decreasing the photoactivity of the MoO_3_/MoS_2_ composite. The aforementioned ions deactivate the catalyst due to the leaching of anions resulting in lack of formation of e^−^/h^+^ pairs during photocatalysis. Because the surface of MoO_3_/MoS_2_ is negatively charged, it interacts with positively charged heavy metals through electrostatic interaction. MoO_3_/MoS_2_ exhibits high adsorption ability; as a results of initial fast adsorption process of Sb^+5^, it was localized on active sites of MoO_3_/MoS_2_ nanocomposite. This process results in high photocatalytic degradation. Due to forming core-shell structure, the light absorption of MoO_3_/MoS_2_ sample in ultraviolet and visible regions increased significantly. As the result, MoO_3_/MoS_2_ composite displays excellent photocatalytic activity under simulated sunlight irradiation. Since no data with Sb^+5^ yield was detected in the recent literature using MoO_3/_MoS_2-E_ composite, the adsorption and photocatalytic yields of the other metals, dyes, and organochlorinated compounds were correlated with our removal. As aforementioned in the introduction section, the Sb^+5^ adsorption and photooxidatıon yields found with this study were relatively higher than Pb^+2^, Au^+3^, Pb^+2^, Cd^+2^ 2, 4, 6-trichlorophenol, RhB a methylene blue dyes [4, 5, 10–17 ].

## 5. Conclusion

Since Sb^+5^ and its compounds are considered to be the first pollutants in aquatic environments in the world and they are found at high levels in water, the treatment of Sb-contaminated wastewater has become more important today. Therefore, in this study, the MoO_3_/MoS_2-E_ composite was produced under laboratory conditions to remove Sb^+5^ from a crude petrochemical wastewater by the photoelectrocatalysis and adsorption process. MoO_3_/MoS_2-E_ composite was generated under laboratory conditions to remove Sb^+5^ from a raw petrochemical wastewater via photoelectrocatalysis process. An Ag/AgCl in saturated KCl, and a Pt electrode was found to be very effective in the removal of Sb^+5^. pH is a very important factor in the photocatalysis of Sb^+5^. For maximum removal of 10 mg/L initial Sb^+5^ concentration (E = 93%), the optimized operational conditions were as follows: 1.20 mg/L MoO_3_/MoS_2-E_ composite concentration, 0.10 mg/L MoO_3_ concentration, 20 min photoelectrocatalysis time, at pH = 9, at HCO_3_^−^, PO_4_^−3^, and SiO_3_^−2^ concentrations of 0.7 and 0.9 mg/L, respectively, at pH = 9.0 and 6.0, respectively, and at a simulated sunlight power of 15 mW/m^2^. The adsorption yield was found to be lower (80%) for the aforementioned operational conditions compared to photocatalysis.

## Figures and Tables

**Figure 3a:**
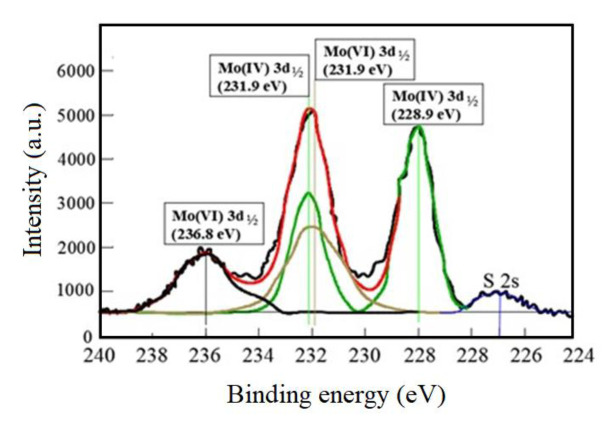
XPS spectra of the Mo 3d in the MoS_2-E_ exposed to the photocatalytic mechanism.

**Figure 3b:**
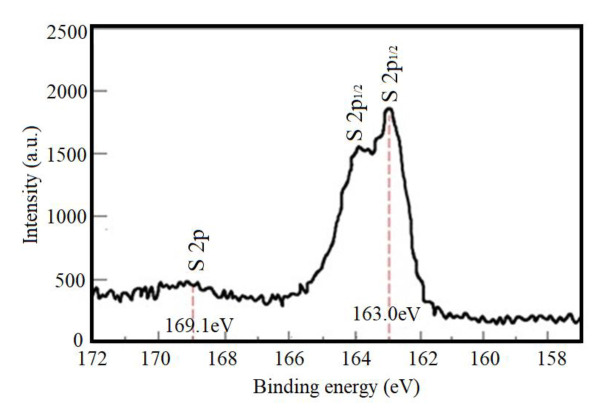
XPS spectra of S and 2p in the MoS_2-E_.

**Figure 3c:**
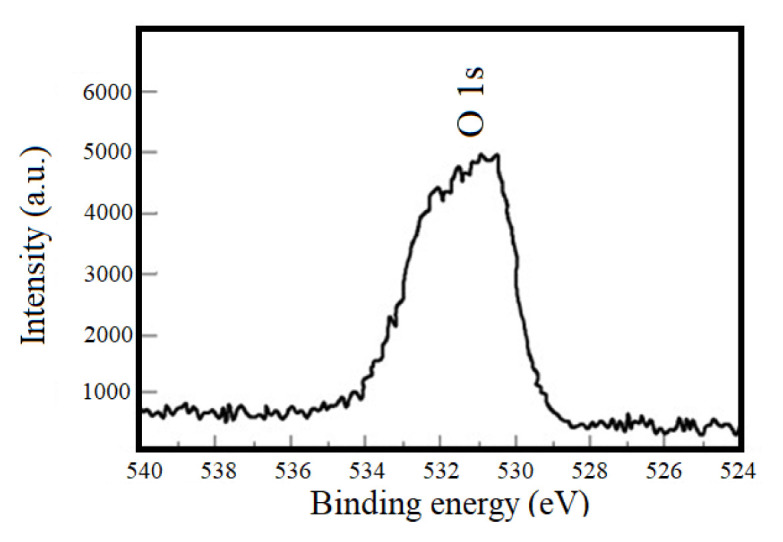
XPS spectra of O1 in the MoS_2-E_.

**Table 1:** Effect of increasing MoO_3_/MoS_2-E_ composite concentrations on the adsorption yields and adsorption capacity (q_e_) of Sb^+5^ at an initial 10 mg/L Sb^+5^ concentration after 20 min contacting time.

MoO_3_/MoS_2-E_ composite concentration (mg/L)	Sb^+5^ concentration (mg/L)	Sb^+5^ removal efficiency (%)	Sb^+5^ concentration (mg/L) after adsorption	Adsorbed Sb^+5^ concentration (mg/L)	q_e_ (mg/g)
0.01	10	10	9	1.0	1.7
0.06	10	20	8	2.0	2.1
0.50	10	40	6	4.0	2.9
1.20	10	80	2	8.0	4.2
3.00	10	80	2	8.0	4.2
6.00	10	80	2	8.0	4.2

**Table 2:** Effect of increasing MoO_3_/MoS_2-E_ composite concentrations on the photoelectrocatalytic removal of Sb^+5^ at an initial 10 mg/L Sb^+5^ concentration at a sunlight power of 15 mW/m^2^ after 20 min photooxidation.

MoO_3_/MoS_2-E_ composite concentration (mg/L)	Sb^+5^ concentration (mg/L)	Sb^+5^ removal efficiency (%)	Sb^+5^ concentration (mg/L) after photoelectrocatalysis	Photocatalyzed Sb^+5^ concentration (mg/L)
0.01	10	20	8.00	2.0
0.06	10	35	6.50	3.5
0.50	10	50	5.00	5.0
1.20	10	93	0.70	9.3
3.00	10	93	0.70	9.3
6.00	10	93	0.70	9.3

**Table 3:** Effects of increasing MoO_3_ concentrations in the 1.20 mg/L MoO_3_/MoS_2-E_ composite concentration on the removal of initial 10 mg/L Sb^+5^ concentration at a sunlight power of 15 mW/m^2^ after 20 min photooxidation.

MoO_3_ concentration (mg/L)	Sb^+5^ concentration (mg/L)	Sb^+5^ removal efficiency (%)	Sb^+5^ concentration (mg/L) after photoelectrocatalysis	Remaining Sb^+5^ concentration (mg/L)
0.05	10	45	5.5	4.5
0.08	10	60	4.0	6.0
0.10	10	92	8.0	2.0
0.15	10	92	8.0	2.0
0.20	10	92	8.0	2.0
0.30	10	92	8.0	2.0

**Table 4:** Effect of photooxidation times on the photoelectrocatalytic removal of 10 mg/L Sb^+5^ concentration at 1.20 mg/L MoO_3_/MoS_2-E_ composite concentration at 0.10 mg/L MoO_3_ concentration at a sunlight power of 15 mW/m^2^ after 20 min photooxidation.

MoO_3_/MoS_2-E_ composite Concentration (mg/L)	Sb^+5^ concentration (mg/L)	Time (min)	Sb^+5^ removal efficiency (%)	Sb^+5^ concentration (mg/L) after photoelectrocatalysis	Remaining Sb^+5^ concentration (mg/L)
1.20	10	5.0	30	7.0	3.0
1.20	10	10	60	6.0	4.0
1.20	10	20	93	9.3	0.7
1.20	10	60	93	9.3	0.7
1.20	10	80	93	9.3	0.7

**Table 5:** Effect of sunlight power on the photoelectrocatalycal removal Sb^+5^.

MoO_3_/MoS_2-E_ composite concentration (mg/L)	Sb^+5^ concentration (mg/L)	MoO_3_ concentration (mg/L)	Sunlight power (W/m^2^)	Sb^+5^ removal efficiency (%)	Remaining Sb^+5^ concentration (mg/L) after photoelectrocatalysis
1.20	10	0.10	2.0	70	3.0
1.20	10	0.10	15	88	1.2
1.20	10	0.10	26	98	0.2

**Table 6:** Effect of pH on the photoelectrocatalytical removal of 10 mg/L Sb^+5^ at 1.20 mg/L MoO_3_/MoS_2-E_ composite concentration at 0.10 mg/L MoO_3_ concentration during increasing photooxidation times.

MoO_3_/MoS_2-E_ composite concentration (mg/L)	MoO_3_ concentration(mg/L)	Sb^+5^ concentration (mg/L)	Time (min)	Sb^+5^ removal efficiency (%)	pH
1.20	0.10	10	3.0	5.0	4
1.20	0.10	10	7.0	9.0	
1.20	0.10	10	10	36	
1.20	0.10	10	15	42	
1.20	0.10	10	20	45	
1.20	0.10	10	3.0	8.0	5
1.20	0.10	10	7.0	12	
1.20	0.10	10	10	18	
1.20	0.10	10	15	33	
1.20	0.10	10	20	47	
1.20	0.10	10	3.0	11	6
1.20	0.10	10	7.0	27	
1.20	0.10	10	10	44	
1.20	0.10	10	15	61	
1.20	0.10	10	20	72	
1.20	0.10	10	3.0	13	7
1.20	0.10	10	7.0	18	
1.20	0.10	10	10	47	
1.20	0.10	10	15	62	
1.20	0.10	10	20	74	
1.20	0.10	10	3.0	20	8
1.20	0.10	10	7.0	41	
1.20	0.10	10	10	62	
1.20	0.10	10	15	70	
1.20	0.10	10	20	81	
1.20	0.10	10	3.0	21	9
1.20	0.10	10	7.0	28	
1.20	0.10	10	10	67	
1.20	0.10	10	15	83	
1.20	0.10	10	20	97	

**Table 7:** Effects increasing NO_3_^−^, Cl^−^, O_3_^2−^, SO_4_^2−^, PO_4_^3−^, and SiO_3_^2−^ ion concentrations on the of 10 mg/L Sb^+5^ at 1.20 mg/L MoO_3_/MoS_2-E_ composite concentration at 0.10 mg/L MoO_3_ concentration after 20 min photooxidation of Sb^+5^ for pH 6 and 9.

Ion types and photooxidation	Ion concentrations ( mg/L) at pH = 6.0			Ion concentrations ( mg/L) at pH = 9.0		
NO_3_^−^ (M)	0	0.5	0.7	0	0.5	0.7
Sb^+5^ Photooxidation removal percentage (%)	72	70	69	81	80	78
Cl^−^ (M)	0	0.5	0.7	0	0.5	0.7
Sb^+5^ Photooxidation removal percentage (%)	72	70	68	81	80	76
CO_3_^2−^ (M)	0	0.5	0.7	0	0.5	0.7
Sb^+5^ Photooxidation removal percentage (%)	72	70	70	81	80	80
SO_4_^2−^_(M),_	0	0.5	0.7	0	0.5	0.7
Sb^+5^ Photooxidation removal percentage (%)	72	70	68	81	80	76
PO_4_^3−^ (M)	0	0.5	0.7	0	0.5	0.7
Sb^+5^ Photooxidation removal percentage (%)	72	78	79	81	83	84
SiO_3_^2− (M)^	0	0.5	0.7	0	0.5	0.7
Sb^+5^ Photooxidation removal percentage (%)	72	75	78	81	86	87

## References

[1] Mubarak H Chai L-Y Mirza N Yang Z-H Pervez A Antimony (Sb)–Pollution and Removal Techniques–Critical Assessment of Technologies Toxicological and Environmental Chemistry 2015 1 37 10.1080/02772248.2015.1095549

[2] Yan G Mao L Jiang B Chen X Gao Y The source apportionment, pollution characteristic and mobility of Sb in roadside soils affected by traffic and industrial activities Journal of Hazardous Materials 2019 121352 10.1016/j.jhazmat.2019.121352 31629593

[3] Anjum A Datta M Adsorptive Removal of Antimony (III) Using Modified Montmorillonite: A Study on Sorption Kinetics J 2012 Journal of Analytical Sciences, Methods and Instrumentation 2012 02 03 167 175 10.4236/jasmi.2012.23027.2012

[4] Hwang MJ Han SW Nguyen TB Hong SC Ryu KS Preparation of MoO3/MoS2/TiO2 composites for catalytic degradation of methylene blue Journal of Nanoscience and Nanotechnology 2012 12 7 5884 5891 10.1166/jnn.2012.6302 22966675

[5] Wen Z Jiale D Zhen Q Ruihua H Yi W Construction of MoS2 nanoarrays and MoO3 nanobelts: Two efficient adsorbents for removal of Pb(II), Au(III) and Methylene Blue Journal of Environmental Sciences 2022 111 2022 38 50 10.1016/j.jes.2021.02.031 34949366

[6] Cummins DR Martinez U Sherehiy A Kappera R Martinez-Garcia A Efficient hydrogen evolution in transition metal dichalcogenides via a simple one-step hydrazine reaction Nature Communications 2016 7 1 10 2016 10.1038/ncomms11857 PMC4906413 27282871

[7] Chhowalla M Shin HS Eda G Li LJ Loh KP The chemistry of two-dimensional layered transition metal dichalcogenide nanosheets Nature Chemistry 2013 5 4 263 275 10.1038/nchem.1589 23511414

[8] Chithambararaj A Rajeswari Yogamalar N Bose AC Hydrothermally synthesized h-MoO3 and α-MoO3 Nanocrystals: new findings on crystal-structure-dependent charge transport Crystal Growth and Design 2016 16 4 1984 1995 10.1021/acs.cgd.5b01571

[9] Li H Yu K Tang Z Fu H Zhu Z High photocatalytic performance of a type-II α-MoO3/MoS2 heterojunction: From theory to experiment Physical Chemistry Chemical Physics 2016 18 20 14074 14085 10.1039/c6cp02027e 27156532

[10] Saadati M Akhavan O Fazli H Single-Layer MoS2-MoO3-x Heterojunction nanosheets with simultaneous photoluminescence and Co-Photocatalytic features Catalysts 2021 11 12 1445 10.3390/catal11121445

[11] Zhao W Liu X Yang X Liu C Qian X Synthesis of novel 1T/2H-MoS2 from MoO3 nanowires with enhanced photocatalytic performance Nanomaterials 2020 10 6 1124 10.3390/nano10061124 32517258 PMC7353267

[12] Cui Z Sun Y From tremella-like MoS2 to α-MoO3 nanoplates: sintering synthesis and adsorption properties Micro & Nano Letter 2017 12 9 652 655 10.1049/mnl.2017.0045

[13] Gusain R Kumar N Fosso-Kankeu E Ray SS Efficient Removal of Pb(II) and Cd(II) from Industrial Mine Water by a Hierarchical MoS2/SH-MWCNT Nanocomposite 2019; ACS Omega, acsomega 9b01603 10.1021/acsomega.9b01603 PMC6714537 31497710

[14] Sheng B Ya F Wang Y Wang Z Li Q Pivotal roles of MoS2 in boosting catalytic degradation of aqueous organic pollutants by Fe(II)/PMS Chemical Engineering Journal 2019 12 1989 10.1016/j.cej.2019.121989

[15] Chandrabose G Dey A Gaur SS Pitchaimuthu S Jagadeesan H Removal and degradation of mixed dye pollutants by integrated adsorption-photocatalysis technique using 2-D MoS2/TiO2 nanocomposite Chemosphere 2021 279 130467 10.1016/j.chemosphere.2021.130467 33857651

[16] Li Z Cao F Wang L Chen Z Ji X A novel ternary MoS2/MoO3/TiO2 composite for fast photocatalytic degradation of rhodamine B under visible-light irradiation New Journal of Chemistry 2020 44 537 542 10.1039/c9nj04107a

[17] Chen J Liao Y Wan X Tie S Zhang B A high performance MoO3/MoS2 porous nanorods for adsorption and photodegradation of dye Journal of Solid State Chemistry 2020 291 121652 10.1016/j.jssc.2020.121652

[18] Zhu C Xu Q Liu W Ren Y CO 2-assisted fabrication of novel heterostructures of h-MoO3/1T-MoS2 for enhanced photoelectrocatalytic performance Applied Surface Science 2017 425 56 62 10.1016/j.apsusc.2017.06.248

[19] Sarojam P APP_Metalsin Wastewater Perkin Elmer Instruments 11 2010 [Online]. http://www.perkinelmer.co.uk/labsolutions/resources/docs/APPMetalsinWastewater

[20] Ghosal PS Gupta AK Determination of thermodynamic parameters from Langmuir isotherm constant-revisited Journal of Molecular Liquids 2017 225 137 146 10.1016/j.molliq.2016.11.058

[21] Hu H Deng C Xu J Zhang K Sun M Metastable h-MoO3 and stable α-MoO3 microstructures: controllable synthesis, growth mechanism and their enhanced photocatalytic activity Journal of Experimental Nanoscience 2015 10 17 1336 1346 10.1080/17458080.2015.1012654

[22] Tang G Wang Y Chen W Tang H Li C Hydrothermal synthesis and characterization of novel flowerlike MoS 2 hollow microspheres Materials Letters 2013 100 15 18 10.1016/j.matlet.2013.02.103

[23] Zhang X Huang X Xue M Ye X Lei W Hydrothermal synthesis and characterization of 3D flower-like MoS2 microspheres Materials Letters 2015 148 67 70 10.1016/j.matlet.2015.02.027

[24] Zhang X Song X Gao S Xu Y Cheng X Facile synthesis of yolk-shell MoO2 microspheres with excellent electrochemical performance as a Li-ion battery anode Journal of Materials Chemistry A 2013 1 23 6858 6864 10.1039/c3ta10399d

[25] Kumar V Sumboja A Wang J Bhavanasi V Nguyen VC Topotactic phase transformation of hexagonal MoO3 to layered MoO3-II and its two-dimensional (2D) nanosheets Chemistry of Material 2014 26 19 5533 5539 10.1021/cm502558t

[26] Li C Zhang D Peng J Li X The effect of pH, nitrate, iron (III) and bicarbonate on photodegradation of oxytetracycline in aqueous solution Journal of Photochemistry and Photobiology A: Chemistry 2018 356 239 247 10.1016/j.jphotochem.2018.01.004

[27] Qi Y Xu Q Wang Y Yan B Ren Y CO2-Induced Phase Engineering: Protocol for Enhanced Photoelectrocatalytic Performance of 2D MoS2 Nanosheets ACS Nano 2016 10 2 2903 2909 10.1021/acsnano.6b00001 26840941

[28] Yin Y Han J Zhang Y Zhang X Xu P Contributions of phase, sulfur vacancies, and edges to the hydrogen evolution reaction catalytic activity of porous molybdenum disulfide nanosheets Journal of the American Chemical Society 2016 138 25 7965 7972 10.1021/jacs.6b03714 27269185

[29] Rabalais JW Colton RJ Guzman AM Trapped electrons in substoichiometric MoO3 observed by X-ray electron spectroscopy Chemical Physics Letters 1974 29 1 131 133 10.1016/0009-2614(74)80149-130

[30] Fleisch TH Zajac GW Schreiner JO Mains GJ An XPS study of the UV photoreduction of transition and noble metal oxides Applied Surface Science 1986 26 4 488 497 10.1016/0169-4332(86)90120-0

[31] Luther JM Jain PK Ewers T Alivisatos AP Localized surface plasmon resonances arising from free carriers in doped quantum dots Nature Materials 2011 10 5 361 366 10.1038/nmat3004 21478881

[32] Manthiram K Alivisatos AP Tunable localized surface plasmon resonances in tungsten oxide nanocrystals Journal of American Chemical Society 2012 134 9 3995 3998 10.1021/ja211363w 22332881

[33] Wang X Zhuang J Peng Q Li Y A general strategy for nanocrystal synthesis Nature 2005 437 7055 121 124 10.1038/nature03968 16136139

[34] Huang P Lin J Li Z Hu H Wang K A general strategy for metallic nanocrystals synthesis in organic medium Chemical Communications 2010 46 26 4800 4802 10.1039/c0cc00307g 20502783

[35] Gao X Hu M Su J Fu Y Yang J Changes in the composition, structure and friction property of sputtered MoS 2 films by LEO environment exposure Applied Surface Science 2015 330 30 38 10.1016/j.apsusc.2014.12.175

[36] Eda G Yamaguchi H Voiry D Fujita T Chen M Photoluminescence from Chemically Exfoliated MoS2 Nano Letter 2011 11 5111 5116 10.1021/nl201874w 22035145

[37] Acerce M Voiry D Chhowalla M Metallic 1T phase MoS2 nanosheets as supercapacitor electrode materials National Nanotechnol 2015 10 4 313 318 10.1038/nnano.2015.40 25799518

[38] Sheng B Liu J Li Z Wang M Zhu K Effects of excess sulfur source on the formation and photocatalytic properties of flower-like MoS2 spheres by hydrothermal synthesis Materials Letters 2015 144 153 156 10.1016/j.matlet.2015.01.056

[39] Garg UK Kaur MP Garg VK Sud D Removal of hexavalent chromium from aqueous solution by agricultural waste biomass Journal of Hazardous Materials 2007 140 1–2 60 68 10.1016/j.jhazmat.2006.06.056 16879918

[40] Chowdhury RR Charpentier PA Ray MB Photodegradation of 17β-estradiol in aquatic solution under solar irradiation: Kinetics and influencing water parameters Chemistry 2011 219 1 67 75 10.1016/j.jphotochem.2011.01.019

[41] Prado AGS Bolzon LB Pedroso CP Moura AO Costa LL Nb2O5 as efficient and recyclable photocatalyst for indigo carmine degradation Applied Catalysis B: Environmental 2008 82 3–4 219 224 10.1016/j.apcatb.2008.01.024

[42] Qi P Pichler T Sequential and simultaneous adsorption of Sb(III) and Sb(V) on ferrihydrite: Implications for oxidation and competition Chemosphere 2016 145 55 60 10.1016/j.chemosphere.2015.11.057 26688239

[43] Nie M Yang Y Zhang Z Yan C Wang X Degradation of chloramphenicol by thermally activated persulfate in aqueous solution Chemical Engineering Journal 2014 246 373 382 10.1016/j.cej.2014.02.047

[44] Watkins R Weiss D Dubbin W Peel K Coles B Arnold T Investigations into the kinetics and thermodynamics of Sb(III) adsorption on goethite (a-FeOOH) Journal Colloid Interface Science 2006 303 639 646 10.1016/j.jcis.2006.08.044 16989849

[45] Wilson NJ Craw D Hunter K Antimony distribution and environmental mobility at an historic antimony smelter site Environmental pollution 2004 129 257 266 10.1016/j.envpol.2003.10.014 14987811

[46] Xi JH He MC Lin CY Zhang P Hu LJ Adsorption of antimony (III) on montmorillonite, kaolinite and goethite: effect of pH and ionic strength Environmental Chemical 2009 28 54 57

[47] Ambe S Adsorption kinetics of antimony(V) ions onto alpha Fe2O3 surfaces from an aqueous solution Langmuir 1987 3 489 493 10.1021/la00076a009

[48] Thanabalasingam P Pickering WF Specific sorption of antimony(III) by the hydrous oxides of Mn, Fe, and Al Water Air Soil Pollution 1990 49 175 185 10.1007/BF00279519

[49] Brox B Olefjord I ESCA studies of MoO2 and MoO3 Surface and Interface Analysis 1988 13 3 6

[50] Addou R Colombo L Wallace RM Surface defects on natural MoS2 ACS Applied Materials & Interfaces 2015 7 11921 11929 25980312 10.1021/acsami.5b01778

[51] Lukowski MA Daniel AS Meng F Forticaux A Li L Enhanced hydrogen evolution catalysis from chemically exfoliated metallic MoS2 nanosheets Journal of the American Chemical Society 2013 135 10274 10277 23790049 10.1021/ja404523s

[52] He Z Liu R Liu H Qu J Adsorption of Sb(III) and Sb(V) on freshly prepared ferric hydroxide (FeOxHy) Environmental Engineering Science 2015 32 2 95 102 10.1089/ees.2014.0155 25741175 PMC4322960

[53] Xi J He M Lin C Adsorption of antimony(III) and antimony(V) on bentonite: Kinetics, thermodynamics and anion competition Microchemical Journal 2011 97 1 85 91 10.1016/j.microc.2010.05.017

[54] Ilgen AG Trainor TP Sb(III) and Sb(V) sorption onto al-rich phases: Hydrous al oxide and the clay minerals kaolinite KGa-1b and oxidized and reduced nontronite NAu-1 Environmental Science & Technology 2012 46 2 843 851 10.1021/es203027v 22136137

[55] Deng RJ Jin CS Ren BZ Hou BL Hursthouse A The Potential for the Treatment of Antimony-Containing Wastewater by Iron-Based Adsorbents Water 2017 9 10 794 10.3390/w9100794

[56] Yang XZ Study on Adsorption of Antimony(III) from Aqueous Solution Using Ggraphene Oxide and It’s Magnetite Composites Master’s Thesis Hunan University Changsha, China 2015 1 115

[57] Zhu Y Wang Y Chen Z Qin L Yang L Visible light induced photocatalysis on CdS quantum dots decorated TiO2 nanotube arrays Applied Catalysis A: General 2015 498 159 166 10.1016/j.apcata.2015.03.035

[58] Zhang Z Cong L Yu Z Qu L Qian M FeNiMo trimetallic nanoparticles encapsulated in carbon cages as efficient hydrogen evolution reaction electrocatalysts Materials Advances 2020 1 1 54 60 10.1039/d0ma00065e

[59] Hu XX Study on the performance and mechanism of the removal of Antimony from mine wastewater by a new type of Fe-Cu binary oxide Master’s Thesis Hunan University of Science and Technology Xiangtan, China 2016 1 73

[60] Santiago DE Araña J González-Díaz O Alemán-Dominguez ME Acosta-Dacal AC Effect of inorganic ions on the photocatalytic treatment of agro-industrial wastewaters containing imazalil Applied Catalysis B: Environmental 2014 156–157 284 292 10.1016/j.apcatb.2014.03.022

[61] Duo L Surface Interactions of Layered Chalcogenides in Covalent Functionalization and Metal Adsorption Arizona State Univer-Pro Quest Dissertations Publishing 2019 22589360

[62] Xi J He M Lin C Adsorption of antimony (V) on kaolinite as a function of pH, ionic strength and humic acid Environmental Earth Science 2010 60 715 722 10.1007/s12665-009-0209-z

[63] Bodzek M Rajca M Photocatalysis in the treatment and disinfection of water. Part I. Theoretical backgrounds Ecological Chemistry and Engineering 2012 19 4 489 512 10.2478/v10216-011-0036-5

[64] Zheng X Shen ZP Shi L Cheng R Yuan DH Photocatalytic Membrane Reactors (PMRs) in Water Treatment: Configurations and Influencing Factors Catalysts 2017 7 8 224 10.3390/catal7080224

[65] Rizzo L Koch J Belgiorno V Anderson MA Removal of methylene blue in a photocatalytic reaktör using polymethylmethacrylate supported TiO2 nanofilm Desalination 2007 211 1 9

[66] McLoughlin OA Ibáñez PF Gernjak W Rodríguez SM Gill LW Photocatalytic disinfection of water using low cost compound parabolic collectors Solar Energy 2004 77 5 625 633 10.1016/j.solener.2004.05.017

[67] Filella M Belzile N Chen YW Antimony in the environment: a review focused on natural waters I. Occurrence Earth-Science Reviews 2002 57 125 176

[68] Liu XW Chen JH Chen JQ Removal of antimony from water by supported iron-zircon bimetal oxide polymeric anion exchange resin Adsorption and Ion Exchange 2016 32 244 252

[69] Wang HW Li XY Li WH Sun Y Effects of pH and complexingagents on Sb(V) adsorption onto binessite and ferrihydrite surface 2017 38 180 187 10.13227/j.hjkx.201606165 29965045

[70] Wang S Zhang D Li B Zhang C Du ZG Ultrastablein-plane 1T-2H MoS2 heterostructures for enhanced hydrogen evolution reaction Advanced Energy Materials 2018 8 1801345 10.1002/aenm.201801345

